# Hematopoietic stem cell transplantation for pediatric patients with non-anaplastic peripheral T-cell lymphoma. An EBMT pediatric diseases working party study

**DOI:** 10.1038/s41409-024-02226-1

**Published:** 2024-02-08

**Authors:** Olga Moser, Maud Ngoya, Jacques-Emmanuel Galimard, Arnaud Dalissier, Jean Hugues Dalle, Krzysztof Kalwak, Wilhelm Wössmann, Birgit Burkhardt, Marc Bierings, Marta Gonzalez-Vicent, Lucía López Corral, Karin Mellgren, Andishe Attarbaschi, Jean Henri Bourhis, Kristina Carlson, Selim Corbacioglu, Katarzyna Drabko, Mikael Sundin, Jacek Toporski, Gunnar Cario, Udo Kontny

**Affiliations:** 1 University Hospital RWTH Aachen, Department of Pediatric Hematology, Oncology, and Stem Cell Transplantation, Aachen, Germany; 2 EBMT Paris Office, Hôpital Saint Antoine, Paris, France; 3 Hôpital Robert Debre Pediatric Hematology and Immunology Department, GHU APHP Nord Université Paris Cité, Paris, France; 4 Department of Pediatric Hematology, Oncology and Bone Marrow Transplantation, Wroclaw Medical University, Wroclaw, Poland; 5 University Medical Center Hamburg Eppendorf, Pediatric Hematology and Oncology, Hamburg, Germany; 6 Pediatric Hematology, Oncology and BMT, University Hospital Münster, Münster, Germany; 7 Princess Maxima Center/ University Hospital for Children (WKZ), Utrecht, The Netherlands; 8 Niño Jesus Children’s Hospital, Stem cell transplant unit, Madrid, Spain; 9 Hematology Department. Hospital Universitario de Salamanca (Spain), IBSAL, CIBERONC. Centro de Investigación del Cáncer-IBMCC (USAL-CSIC), Salamanca, Spain; 10 Sahlgrenska University Hospital, Department of Pediatric Oncology, Göteborg, Sweden; 11 St. Anna Children’s Hospital. Department of Pediatric Hematology and Oncology, Medical University of Vienna, Vienna, Austria; 12 St. Anna Children’s Cancer Research Institute, Vienna, Austria; 13 Département d’Hématologie, Institut Gustave Roussy, Villejuif, France; 14 University Children’s Hospital Dept. of Women’s & Children’s Health, Uppsala, Sweden; 15 University Hospital Regensburg, Paediatric Haematology, Oncology and Stem Cell Transplantation, Regensburg, Germany; 16 Medical University of Lublin, Dept. Pediatric Hematology, Oncology, and Transplantology, Lublin, Poland; 17 Karolinska University Hospital Children’s Hospital, Paediatric Haematology, Stockholm, Sweden; 18 University Medical Center Schleswig-Holstein Kiel, División of Stem Cell Transplantation and Immunotherapy, Kiel, Germany

**Keywords:** Cancer, Risk factors

## Abstract

Peripheral T-cell lymphomas (PTCL) other than anaplastic large-cell lymphoma are rare in children, and the role of hematopoietic stem cell transplantation (HSCT) has not been clarified yet. In a retrospective analysis of registry-data of the European Society for Blood and Marrow Transplantation we analyzed 55 patients aged < 18 years who received allogeneic (*N* = 46) or autologous (*N* = 9) HSCT for PTCL. Median age at HSCT was 13.9 years; 33 patients (60%) were in first remission, and 6 (19%) in progression at HSCT. Conditioning was myeloablative in 87% of the allogeneic HSCTs and in 27 (58.7%) based on total body irradiation. After allogeneic HSCT the 5-year overall- and progression-free survival was 58.9% (95% CI 42.7–71.9) and 52.6% (95% CI 36.8–66.1), respectively. 5-year relapse incidence was 27.6% (95% CI 15.1–41.6), the non-relapse mortality rate was 19.8% (95% CI 9.7–32.6). Five of the six patients with progression at HSCT died. Seven of nine patients after autologous HSCT were alive and disease-free at last follow-up. Our data suggest a role of allogeneic HSCT in consolidation-treatment of patients with high-risk disease, who reach at least partial remission after primary- or relapse-therapy, whereas patients with therapy-refractory or progressive disease prior to transplantation do not profit from HSCT.

## Introduction

Peripheral T-cell lymphoma (PTCL) is a rare and heterogenous group of Non-Hodgkin-Lymphoma (NHL) [1], accounting for 0.9–1.8% of childhood NHL [2–4]. Distribution of PTCL-subtypes in pediatrics differs from reports in adults [5]. In children, most common is PTCL non-otherwise specified (PTCL-NOS), followed by extranodal natural-killer/T-cell lymphoma (ENKTCL)-nasal type, hepatosplenic T-cell lymphoma (HSTCL), subcutaneous-panniculitis-like T-cell lymphoma (SPTCL), and angioimmunoblastic T-cell lymphoma (AITL). Because of the low incidence there is limited data regarding the optimal treatment for pediatric PTCL. Recent reports show major differences in the gene expression profile and somatic mutations between adult and pediatric PTCL indicating different biology of the diseases [5, 6]. Accordingly, children with PTCL might benefit from different treatment strategies than adults. The 5-years overall survival (OS) rates of approximately 55% are inferior compared to other NHL-entities in children, and highly subgroup-dependent, ranging from 83% for SPTCL to 16% for HSTCL [2–4, 7–10]. Patients presenting with advanced-stage disease show worse outcome. In addition, patients with preexisting conditions such as primary or secondary immunodeficiency (up to 25% of children with PTCL) have a dismal outcome with OS around 30% [7]. The role of autologous/allogeneic hematopoietic stem-cell transplantation (HSCT) in the treatment of pediatric PTCL is still unclear. Therefore, we performed a retrospective analysis of HSCT-outcomes for children with PTCL based on registry data of the European Society for Blood and Marrow Transplantation (EBMT).

## Patients and methods

Patients aged < 18 years at first transplantation for PTCL according to the WHO-classification [1], except of ALK-positive and negative anaplastic large-T-cell lymphoma (ALCL), transplanted between 1995 and 2015, who received allogeneic HSCT (allo-HSCT) or autologous HSCT (ASCT) were included in the analysis.

We reviewed the data of all patients fulfilling the inclusion criteria from centers reporting to the database of the EBMT. In addition, a specifically designed questionnaire was sent to all participating centers for information that was not available from the database (see supplementary material). Analyses were conducted in accordance with the Declaration of Helsinki, after signed informed consent of the legal guardians had been obtained.

### Statistic methods

Patients, donors and HSCT characteristics were described using medians and interquartile ranges (IQR) for quantitative variables and percentages for categorical variables.

The study primary endpoints were OS and progression-free survival (PFS). Secondary endpoints were relapse incidence (RI), non-relapse mortality (NRM). OS was defined as the time from transplantation to death, regardless of cause. PFS was calculated as time from transplant to relapse, progression or death from any other cause. Relapse was defined as progressive lymphoma after HSCT or lymphoma recurrence after complete remission. NRM was defined as death without relapse or progression.

OS and PFS were calculated using the Kaplan-Meier estimates. Cumulative incidence functions were used to estimate the endpoints for RI, NRM, acute/chronic GVHD, and neutrophil engraftment to accommodate for competing risks. The follow-up period for patients was calculated using reverse Kaplan-Meier estimates.

Statistical analyses were performed with R 4.0.2 [R Core Team (2017). R: A language and environment for statistical computing; R Foundation for Statistical Computing, Vienna, Austria] software packages.

## Results

**Fig. 1:**
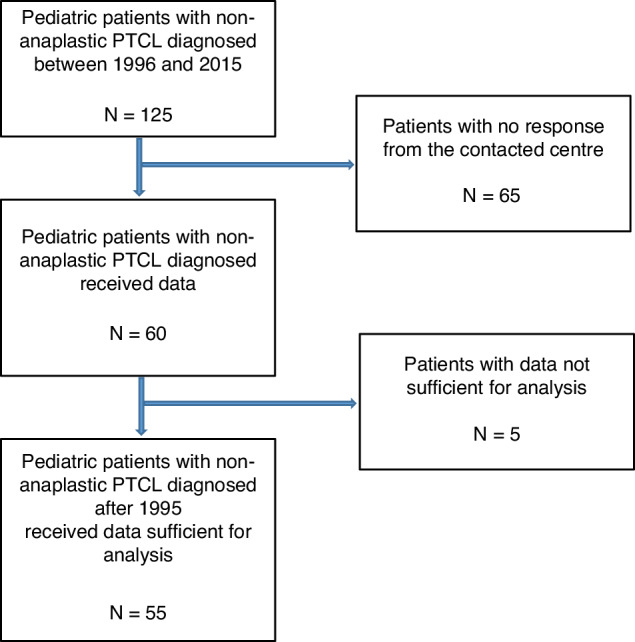
Study population chart. Numbers of patients screened from the EBMT-database for inclusion in the present study.

Out of 125 patients < 18 years diagnosed with PTCL who were registered to the EBMT-database and transplanted between 1995 and 2015, we obtained sufficient data about HSCT in 55 patients (Fig. 1). Thirty-eight patients had PTCL-NOS, 7 HSTCL, 4 AITL, 3 ENKTCL, 2 SPTCL, and 1 systemic EBV-positive T-cell lymphoma of childhood. Median age at HSCT was 13.9 years (range: 2.5–17.9) [IQR 9.73–16.25]; 36 (65.4%) of the patients were male. Of all analyzed patients 4 suffered from a known cancer-predisposing syndrome/primary immunodeficiency, and 4 from secondary immunodeficiency. 42 (91.3%) out of 46 patients with available staging data presented with advanced-stage disease (stage III/IV), and 23 (62.2%) with B-symptoms at first diagnosis. Patient characteristics are depicted in Table 1.

**Table 1:** Patient characteristics.

		Total (*N* = 55)	Allogeneic (*N* = 46)	Autologous (*N* = 9)
Age at diagnosis in years	Median	13	12.6	13.1
	(range)	(0.7–17.6)	(0.7–17.6)	(4.5–17.5)
	[IQR]	[7.6–15.33]	[7.4–15.28]	[8.8–15.9]
Age at HSCT in years	≥ 2–7	9 (16.4%)	8 (17.4%)	1 (11.1%)
	> 7–12	13 (23.6%)	12 (26.1%)	1 (11.1%)
	> 12–< 18	33 (60%)	26 (56.5%)	7 (77.8%)
	Median	13.88	13.9	13.3
	(range)	(2.5–17.9)	(2.5–17.9)	(6.5–17.8)
	[IQR]	[9.73–16.25]	[9.3–16]	[13.1–17.4]
Year of HSCT		1996–2015	1996–2015	1996–2011
Patient sex	Female	19 (34.5%)	18 (39.1%)	1 (11.1%)
	Male	36 (65.5%)	28 (60.9%)	8 (88.9%)
Histologic subtype	PTCL-NOS	38 (69.1%)	30 (65.2%)	8 (88.9%)
	HSTCL	7 (12.7%)	7 (15.2%)	0 (0%)
	AITL	4 (7.3%)	4 (8.7%)	0 (0%)
	ENKTCL	3 (5.5%)	3 (6.5%)	0 (0%)
	SPTCL	2 (3.6%)	1 (2.2%)	1 (11.1%)
	Systemic EBV + TCLC	1 (1.8%)	1 (2.2%)	0 (0%)
Size of the largest mass	< 5 cm	15 (37.5%)	14 (42.4%)	1 (14.3%)
	> 10 cm	5 (12.5%)	5 (15.2%)	0 (0%)
	5–10 cm	8 (20%)	5 (15.2%)	3 (42.9%)
	No mass	6 (15%)	5 (15.2%)	1 (14.3%)
	Not measurable	6 (15%)	4 (12.1%)	2 (28.6%)
	Missing	15	13	2
Stage of disease at diagnosis	I	2 (4.4%)	1 (2.6%)	1 (12.5%)
	II	2 (4.4%)	1 (2.6%)	1 (12.5%)
	III	17 (37.0%)	15 (39.5%)	2 (25%)
	IV	25 (54.4%)	21 (55.3%)	4 (50%)
	Missing	9	8	1
Systemic symptoms	A	14 (37.8%)	10 (33.3%)	4 (57.1%)
	B	23 (62.2%)	20 (66.7%)	3 (42.9%)
	Missing	18	16	2
Known cancer predisposition syndrome/primary immunodeficiency*	No	43 (91.5%)	34 (89.5%)	9 (100%)
	Yes	4 (8.5%)	4 (10.5%)	0 (0%)
	Missing	8	8	0
Acquired immunodeficiency or immunosuppression**	No	46 (92%)	37 (90.2%)	9 (100%)
	Yes	4 (8%)	4 (9.8%)	0 (0%)
	Missing	5	5	0

*Known primary immunodeficiency patients (Number of patients): Nijmegen breakage syndrome (2), heterozygotic mutation in UNC13D gene (1), congenital EBV-infection (1).

**Patients with secondary immunodeficiency (number of patients): autoimmune hepatitis treated with Azathioprin (2010-2014) (1), Azathioprin-treatment (1), earlier leukemia treatment including chemo- and radiotherapy (1), Still-disease (1).

*AITL* Angioimmunoblastic T-cell lymphoma, *ENKTCL* Extranodal natural-killer/T-cell lymphoma, *HSTCL* Hepatosplenic T-cell lymphoma, *PTCL-NOS* PTCL non-otherwise specified, *SPTCL* subcutaneous-panniculitis-like T-cell lymphoma, systemic EBV + TCLC: systemic EBV-positive T-cell lymphoma of childhood.

### Treatment prior to HSCT

In primary treatment there was an equal distribution between lymphoblastic lymphoma/T-cell-type- and pulse-B-NHL/ALCL-like chemotherapy (12 versus 10 patients, respectively), as previously described [11–17]. 7 patients received miscellaneous treatment, however there was missing information about primary treatment in 26 patients. Radiotherapy was not part of the primary treatment in most of the patients (97.9%). 26 patients (49.1%) achieved complete remission (CR) after the primary treatment, 12 (22.6%) showed partial response (PR), 6 (11.3%) stable disease (SD), and 9 (17%) progressive/refractory disease (PD), respectively. Information about status after primary treatment was missing in two patients. 28 (53.9%) patients relapsed after the first-line treatment. Before HSCT, 30 patients (55.6%) showed at least one relapse. Of those, 26 (57.8%) proceeded eventually to allo-HSCT, and 4 (44.4%) to ASCT. 24 patients were without relapse prior to HSCT, 19 (42.2%) of them received allo-HSCT, and 5 (55.6%) ASCT, respectively. For one patient information was missing.

### Status at first HSCT

Thirty-three (60%) patients were in CR before the first HSCT, 16 (29.1%) were in PR or very good PR (VGPR). 6 (10.9%) patients showed PD at first HSCT. In our cohort all 7 patients with HSTCL, and 22/38 patients with PTCL-NOS were in CR1/PR1 at HSCT. Conversely, only one of four patients with AITL and one of three patients with ENKTCL were in CR1/PR1 at HSCT. Most of the younger patients (<7 years) achieved CR1/PR1 prior to HSCT (8 out of 9 patients). Patients with stage I/II disease all achieved a CR1/PR1 status, while patients with stage III or IV were in CR1/PR1 in 29% and 68%, respectively. 56.5% of patients presenting with B-symptoms were in CR1/PR1 before HSCT. Table 2 describes status at first HSCT.

**Table 2:** Status at first HSCT.

		Total (*N* = 55)	CR or PR or VGPR = 1 (*N* = 33)	Other (*N* = 22)
Age at HSCT in years	Median	13.9	14.8	13.3
	(range)	(2.5–17.9)	(2.5–17.9)	(3.6–17.7)
	[IQR]	[9.7–16.2]	[7.2–16.7]	[10–15.2]
Age at HSCT in years	0–7	9 (16.4%)	8 (24.2%)	1 (4.6%)
	> 7–12	13 (23.6%)	5 (15.2%)	8 (36.4%)
	> 12–< 18	33 (60%)	20 (60.6%)	13 (59.1%)
Patient sex	Female	19 (34.5%)	10 (30.3%)	9 (40.9%)
	Male	36 (65.5%)	23 (69.7%)	13 (59.1%)
Histologic subtype	PTCL-NOS	38 (69.1%)	22 (66.7%)	16 (72.7%)
	HSTCL	7 (12.7%)	7 (21.2%)	0 (0%)
	AITL	4 (7.3%)	1 (3.0%)	3 (13.6%)
	ENKTCL	3 (5.5%)	1 (3.0%)	2 (9.1%)
	SPTCL	2 (3.6%)	1 (3.0%)	1 (4.6%)
	Systemic EBV + TCLC	1 (1.8%)	1 (3.0%)	0 (0%)
Size of the largest mass	< 5 cm	15 (37.5%)	9 (45%)	6 (42.9%)
	> 10 cm	5 (12.5%)	4 (20%)	1 (7.1%)
	5–10 cm	8 (20%)	4 (20%)	4 (28.6%)
	No mass	6 (15%)	3 (15%)	3 (21.4%)
	Not measurable/missing	21	13	8
Stage of disease at diagnosis	I	2 (4.4%)	2 (7.7%)	0 (0%)
	II	2 (4.4%)	2 (7.7%)	0 (0%)
	III	17 (37.0%)	5 (19.2%)	12 (60%)
	IV	25 (54.4%)	17 (65.4%)	8 (40%)
	Missing	9	7	2
Systemic symptoms	A	14 (37.8%)	11 (45.8%)	3 (23.1%)
	B	23 (62.2%)	13 (54.2%)	10 (77%)
	Missing	18	9	9
Known cancer predisposition syndrome/primary immunodeficiency	No	43 (91.5%)	26 (92.9%)	17 (89.5%)
	Yes	4 (8.5%)	2 (7.1%)	2 (10.5%)
	Missing	8	8	0
Known secondary immunodeficiency or immunosuppression	No	46 (92%)	27 (90%)	19 (95%)
	Yes	4 (8%)	3 (10%)	1 (5%)
	Missing	5	2	3
Disease status at first HSCT	CR/PR/VGPR = 1	33 (60%)	33 (100%)	0
	CR = 1	22	22	0
	PR = 1	8	8	0
	VGPR = 1	3	3	0
	CR/PR/VGPR > 1	16 (29.1%)	0	16 (72.7%)
	CR > 1	11	0	11
	PR > 1	3	0	3
	VGPR > 1	2	0	2
	Progression	6 (10.9%)	0	6 (27.3%)
	Refractory disease	3	0	3
	PD	2	0	2
	Untreated relapse	1	0	1

*AITL* Angioimmunoblastic T-cell lymphoma, *CR* Complete remission, *ENKTCL* Extranodal natural-killer/T-cell lymphoma, *HSTCL* Hepatosplenic T-cell lymphoma, *n.u.* number unknown, *PR* Partial remission, *PTCL-NOS* PTCL non-otherwise specified, *SPTCL* Subcutaneous-panniculitis-like T-cell lymphoma, systemic EBV + TCLC: systemic EBV-positive T-cell lymphoma of childhood, VGPR: very good partial remission.

Patient characteristics are shown for patients transplanted in first remission (CR, VGPR or PR) versus other patients.

### HSCT

Forty-six patients underwent allo-HSCT, and 9 patients ASCT (eight patients with PTCL-NOS and one with SPTCL). All patients diagnosed with HSTCL, AITL, ENKTCL or systemic EBV-positive T-cell lymphoma of childhood, and all patients with a known preexisting condition underwent allo-HSCT (Table 3).

**Table 3:** HSCT-related characteristics.

		Total (*N* = 55)	Allogeneic	Autologous
			(*N* = 46)	(*N* = 9)
Age at HSCT	Median	13.9	13.9	13.3
	(range)	(2.5–17.9)	(2.5–17.9)	(6.5–17.8)
	[IQR]	[9.73–16.25]	[9.3–16]	[13.1–17.4]
Response to primary treatment	Complete remission (CR)	26 (49.1%)	19 (42.2%)	7 (87.5%)
	Partial remission (PR)	12 (22.6%)	12 (26.7%)	0
	Relapse/progression (PD)	9 (17.0%)	8 (17.8%)	1 (12.5%)
	Stable disease (SD)	6 (11.2%)	6 (13.3%)	0
	Missing	2	1	1
Disease status at first HSCT	Complete remission (CR)	33 (60%)	25 (54.4%)	8 (88.9%)
	Partial remission (PR/ VGPR)	16 (29.1%)	16 (34.8%)	0
	Refractory/progressive disease (PD)	6 (10.9%)	5 (10.9%)	1 (11.1%)
Disease status at first HSCT (detailed)	CR/PR/VGPR = 1	33 (60%)	27 (58.7%)	6 (66.7%)
	CR = 1	22	16	6
	PR = 1	8	8	0
	VGPR = 1	3	3	0
	CR/PR/VGPR > 1	16 (29.1%)	14 (30.4%)	2 (22.2%)
	CR > 1	11	9	2
	PR > 1	3	3	0
	VGPR > 1	2	2	0
	Progression	6 (10.9%)	5 (10.7%)	1 (11.1%)
	Refractory disease	3	3	0
	PD	2	1	1
	Untreated relapse	1	1	0
Stem cell source	Bone marrow	24 (43.6%)	24 (52.2%)	0
	Perpheral Blood stem cells	29 (52.7%)	20 (43.5%)	9 (100%)
	Cord blood (CB)	2 (3.6%)	2 (4.4%)	0
Donor type	Autologous	9 (16.4%)	n.a.	9 (100%)
	Syngeneic	1 (1.8%)	1 (2.2%)	
	HLA-identical sibling	13 (23.6%)	13 (28.3%)	
	Other matched family donor	1 (1.8%)	1 (2.2%)	
	MUD (10/10)	17 (30.9%)	17 (37.0%)	
	MMUD (9/10)	5 (9.1%)	5 (10.9%)	
	MMUD (8/10)	1 (1.8%)	1 (2.2%)	
	Haploidentical HSCT	1 (1.8%)	1 (2.2%)	
	Single CB unit-HSCT	2 (3.6%)	2 (4.4%)	
	Unrelated (no HLA typing to check)	5 (9.1%)	5 (10.9%)	
Donor type (combined)	Autologous	9 (16.4%)	n.a.	9 (100%)
	HLA-identical sibling or syngeneic	14 (25.5%)	14 (30.4%)	
	Other matched family donor	1 (1.8%)	1 (2.2%)	
	MUD (10/10)	17 (30.9%)	17 (37.0%)	
	Other unrelated donor	13 (23.6%)	13 (28.3%)	
	Haploidentical SCT	1 (1.8%)	1 (2.2%)	
MAC	No	6 (10.9%)	6 (13.0%)	0
	Yes	49 (89.1%)	40 (87.0%)	9 (100%)
TBI	No	28 (50.9%)	19 (41.3%)	9 (100%)
	Yes	27 (49.1%)	27 (58.7%	0
Radiotherapy other than TBI	No	46 (97.9%)	39 (100%)	7 (87.5%)
	Yes	1 (2.1%)	0	1 (12.5%)
	Missing	8	7	1
Conditioning regimen	BEAM	6 (10.9%)	2 (4.4%)	4 (44.4%)
	Bu-based	12 (21.8%)	10 (21.7%)	2 (22.2%)
	Flu-based	7 (12.7%)	7 (15.2%)	0
	Other without TBI	3 (5.6%)	0	3 (33.3%)
	TBI-Cy	8 (14.6%)	8 (17.4%)	0
	TBI-Cy + ARA-C or Eto or Flu	5 (9.1%)	5 (10.9%)	0
	TBI-Eto	10 (18.2%)	10 (21.7%)	0
	TBI-Eto + Mel/Thio	3 (5.6%)	3 (6.5%)	0
	Other with TBI	1 (1.8%)	1 (2.2%)	0
In vivo T cell depletion	No	16 (36.4%)	16 (36.4%)	
	Yes	28 (63.6%)	28 (63.6%)	
	Missing information	2	2	
Graft manipulation ex vivo	No	50 (92.6%)	42 (93.3%)	8 (88.9%)
	Yes	4 (7.4%)	3 (6.7%)	1 (11.1%)
	Missing information	1	1	0
DLI post HSCT	No	5 (26.3%)	4 (23.5%)	1 (50%)
	Yes	14 (73.7%)	13 (76.5%)	1 (50%)*
	Missing information	36	29	7

Percentage values are given for evaluable patients.

*DLI were given after second allo-HSCT.

*ARA-C* Cytarabine, *BEAM* BCNU (carmustine), etoposide, *ARA-C* melphalan, *Bu* Busulfan, *Cy* Cyclophosphamide, *DLI* Donor lymphocyte infusions, *Eto* Etoposide, *Flu* Fludarabine, *IQR* Interquartile range, *MAC* Myelo-ablative conditioning, *Mel* Melphalan, *MUD* Matched unrelated donor, *MMUD* Mismatched unrelated donor, *Thio* Thiotepa, *TBI* Total body-irradiation.

### Donor and stem cell source

HLA-matched family donors were used in 15 patients (13 identical siblings, 1 syngeneic twin, and 1 other, respectively). Seventeen patients were transplanted from a 10/10 HLA-matched unrelated donor, 5 from a 9/10 unrelated donor, 1 from 8/10 unrelated donor. One patient received haploidentical HSCT, and 2 unrelated single-cord-blood transplantation. In five patients transplanted from unrelated donors there were no records on HLA-typing. Bone marrow (BM), peripheral blood stem cells (PBSC), and cord blood were the stem cell source in 24, 20, and 2 patients for allo-HSCT, respectively. All patients with ASCT received PBSC (Table 3).

### Conditioning regimen

The conditioning regimen varied considerably in the studied population. Myelo-ablative-conditioning (MAC) was used in 49 (89.1%) patients (all ASCT and 87% of the allo-HSCT). Only six patients, who were in remission (CR/PR or VGPR) prior to HSCT received non-myeloablative regimen prior to allo-HSCT.

Total body irradiation (TBI) was part of the conditioning regimen in 27 patients receiving allo-HSCT (58.7%). TBI-based conditioning (12 Gy, 10 Gy, and 6 Gy in 23, 1 and 2 patients, respectively (TBI-dose for one patient missing) comprised either cyclophosphamide or etoposide (Table 3). TBI was preferred over chemotherapy-based conditioning in patients not in first remission (CR1/PR1 or VGPR = 1) prior to HSCT (59.1% vs. 40.9%). In this group TBI/etoposide-based conditioning was used more frequently than TBI/cyclophosphamide-based conditioning (10 vs. 3 patients, respectively). 57.6% of the patients in remission received chemotherapy-based conditioning without TBI.

The most common non-TBI conditioning regimen for allo-HSCT was busulfan-based (in 12 (21.8%) patients). Busulfan-free conditioning were mostly fludarabine-based. Most common conditioning prior to ASCT was BEAM (cytarabine, BCNU, etoposide, melphalan), that was used in 4 patients (Table 3).

In-vivo T-cell depletion was performed in 28 (63.6%) of the allografts, graft manipulation ex-vivo was done in 4 (7.4%) cases (Table 3).

### Outcome after HSCT

**Fig. 2:**
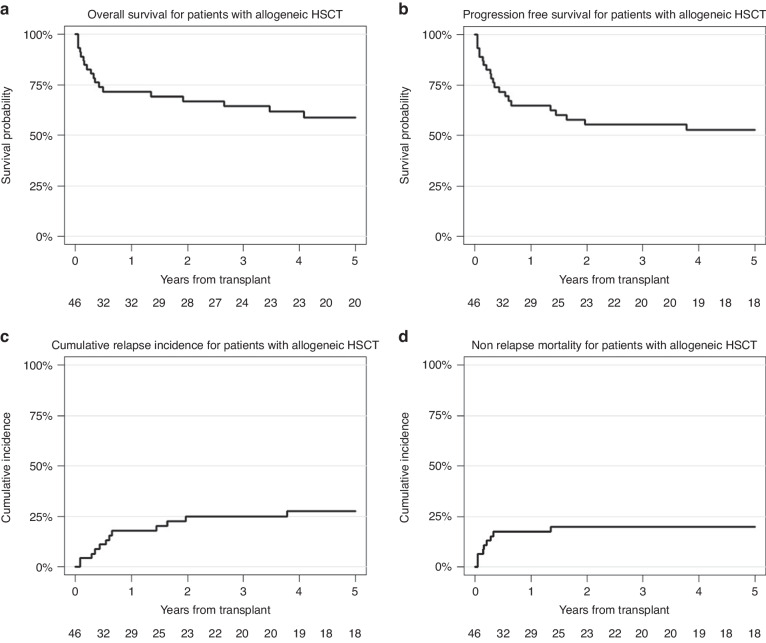
Study population chart Numbers of patients screened from the EBMT-database for inclusion in the present study. Outcome estimates after allogeneic HSCT: **a** Kaplan-Meier estimates of overall survival (OS). The 2-year OS was 66.8% (95% CI 51.1–78.5), the 5-year OS was 58.9% (95% CI 42.7–71.9), respectively. **b** Probability of progression-free survival (PFS): The 2-year PFS was 55.3% (95% CI 39.6-68.5), the 5-year PFS was 52.6% (95% CI 36.8-66.1), respectively. **c** Cumulative relapse incidence (RI): 2-year cumulative RI was 24.9% (95% CI 13.2–38.4), 5-year cumulative RI was 27.6% (95% CI 15.1–41.6), respectively. **d** Estimate of non-relapse mortality (NRM): the 2-year and 5-year NRM was 19.8% (95% CI 9.7–32.6).

With a median follow-up of 6.9 years (95% CI 5.4–8.1) the 5-year OS and PFS was 58.9% (95% CI 42.7–71.9) and 52.6% (95% CI 36.8–66.1), respectively, for patients who received allo-HSCT. The 5-year cumulative RI for patients with allo-HSCT was 27.6% (95% CI 15.1–41.6), whereas the 5-year NRM was 19.8% (95% CI 9.7–32.6), (Fig. 2).

After ASCT, with median follow-up of 9.9 years (95% CI 4.5–14.1), seven of nine patients remained in remission and were alive at last follow-up. One patient transplanted in CR1 relapsed after ASCT, proceeded to allo-HSCT 80 days after the ASCT, subsequently developed extensive chronic GvHD and died of HSCT-related cause. Another patient also died of HSCT-related cause after transplantation in PD-status. (Table 4).

**Table 4:** HSCT-outcome.

		Total (*N* = 55)	Allogeneic	Autologous
			(*N* = 46)	(*N* = 9)
Hematopoetic recovery (engraftment)	Engrafted	52 (94.6%)	43 (93.5%)	9 (100%)
	No engraftment	3 (5.4%)	3 (6.5%)	0
	Missing	0	0	0
Best response within day +100 after first HSCT	Complete remission (CR)	40 (74.1%)	33 (73.3%)	7 (77.8%)
	Patrial remission ( > 50%)	2 (3.7%)	2 (4.4%)	0
	Never in CR	4 (7.4%)	4 (8.9%)	0
	Relapse/progression	1 (1.9%)	0	1 (11.1%)
	Death	5 (9.3%)	4 (8.9%)	1 (11.1%)
	Not evaluated/missing	3 (5.5%)	3 (6.5%)	0
Relapse after the HSCT	No	39 (75%)	31 (72.1%)	8 (88.9%)
	Yes	13 (25%)	12 (27.9%)	1 (11.1%)
	Missing	3	3	0
Survival status at last follow-up	Alive	27 (49.1%)	21 (45.7%)	6 (66.7%)
	Dead	20 (36.4%)	18 (39.1%)	2 (22.2%)
	Lost to follow-up	8 (14.5%)	7 (15.2%)	1 (11.1%)
Main cause of death	HSCT-related	11 (55%)	9 (50%)	2 (100%)
	Relapse/progression	9 (45%)	9 (50%)	0
Acute GvHD	No		18 (40%)	
	Grade I		7 (15.6%)	
	Grade II		11 (24.4%)	
	Grade III		5 (11.1%)	
	Grade IV		2 (4.4%)	
	Present, grade unknown		2 (4.4%)	
	Missing		1	
Chronic GvHD	No		38 (88.4%)	
	Yes		5 (11.6%)	
	Extensive		2 (4.7%)	
	Missing		3	

*GvHD* Graft versus host disease.

Table 5 shows the outcome according to remission status prior to the HSCT. There was no major difference in OS between patients transplanted in first remission (CR1/PR1) versus CR/PR > 1. Of the six patients not in remission prior to the HSCT one patient was treated with a tandem-ASCT and died 73 days after the first ASCT of SCT-related cause. Five patients received allo-HSCT, 4 of them relapsed and died of PTCL.

**Table 5:** Outcome after HSCT according to remission status at HSCT.

		Total	CR/PR = 1	CR/PR > 1	PD
Total		55	33 (60%)	16 (29.1%)	6 (10.9%)
Allo-HSCT		46 (100%)	27 (58.7%)	14 (30.4%)	5 (10.9%)
	alive	27 (58.7%)	16 (59.3%)	10 (71.4%)	1 (20%)
	dead	19 (41.3%)	11 (40.7%)	4 (28.6%)	4 (80%)
	Main cause of death: DOD	10 (52.6%)	4 (36.4%)	2 (50%)	4 (100%)
	Main cause of death: NRM	9 (47.4%)	7 (63.6%)	2 (50%)	0
ASCT		9 (100%)	6 (66.7%)	2 (22.2%)	1 (11.1%)
	alive	7 (77.8%)	5 (83.3%)	2 (100%)	0
	dead	2 (22.2%)	1 (16.7%)	0	1 (100%)
	Main cause of death: DOD	0	0	0	0
	Main cause of death: NRM	2 (100%)	1 (100%)	0	1 (100%)

*CR/PR* *=* *1* First complete/partial remission, *CR/PR* *>* *1* Second or later complete/partial remission, *DOD* Dead of disease, *NRM* Non-relapse mortality, *PD* Progressive disease.

Of the 8 patients with primary- (4) or secondary (4) immunodeficiency, 5 (62.5%) survived, one patient died of infection, two relapsed and died of lymphoma progression (Table 6).

**Table 6:** Characteristics/outcome of patients with preexisting condition.

	Preexisting condition (details)	Status at first HSCT	Relapse after HSCT?	outcome	Cause of death
Primary ID/ Cancer predisposition	4 patients		1 patient	2 dead	
	NBS	CR 1	no	dead	Infection (d 491) (NRM)
	NBS	PR > = 3	yes	dead	DOD
	Mutation UNC 13D gene (heterozygot)	VGPR	no	alive	
	Congenital EBV	PR 2	No	alive	
Secondary ID	4 patients		1 patient	1 dead	
	Autoimmune hepatitis (Azathioprine)	PR1	No	alive	
	Azathioprine	CR1	No	alive	
	Earlier leukemia with irradiation	CR1	no	alive	
	Still disease	Never in CR	yes	dead	DOD

*CR* Complete remission, *DOD* Death of disease (relapse/lymphoma progression), *EBV* Epstein Barr Virus, *ID* Immune deficiency, *NBS* Nijmegen breakage syndrome, *NRM* Non-relapse mortality, *PR* Partial remission, *VGPR* Very good partial remission.

### Engraftment

The cumulative incidence of leukocyte engraftment within 30 days after allo-HSCT was 88.6% (95% CI 73.6–95.4). All patients engrafted after ASCT (Table 4). Of the five patients with no or delayed engraftment, four died of SCT-related causes.

### Relapses after first HSCT and NRM

Relapse was observed in 11 patients after allo-HSCT, and in one patient after ASCT. Ten of them died of lymphoma. NRM was observed in 9 patients after allo-HSCT, and 2 patients after ASCT. There were 3 early deaths without engraftment, and 4 more before day +100 after allo-HSCT. The main causes of death were infections in 5 patients, alone or in combination with veno-occlusive disease (3 patients), and/or GvHD (2 patients). Two patients died of CNS toxicity.

### GvHD

The univariate analysis showed the incidence of acute GvHD grade II-IV within 100 days after allo-HSCT of 41.9% (95% CI 26.9–56.1). The incidence of severe aGvHD (grade III/IV) within 100 days was 16.3% (95% CI 7.1–28.8). The incidence of chronic GvHD was 11.9% (95% CI 4.2–23.8) and the incidence of extensive chronic GvHD was 4.8% (95% CI 0.8–14.6).

## Discussion

In contrast to adult patients, few studies investigated the role of HSCT in treatment of PTCL in the pediatric population. The heterogeneity of the diseases as well as better outcome for most of the pediatric PTCL-subtypes after first-line therapy compared to their adult counterparts, render extrapolating results from adult studies difficult.

In pediatric studies EFS/OS varied considerably between the different subtypes. In the up to now largest survey of pediatric patients with PTCL by the EICNHL and I-BFM Study Group [7] comprising 143 patients, 5-year OS and EFS for all patients was 0.56 ± 0.05 and 0.45 ± 0.05, respectively. Relapse occurred in 27%. Twenty-five patients (17.5%) were chemotherapy-refractory. The subgroup analysis revealed following 5-year OS: PTCL-NOS 56%, ENKTCL 59%, SPTCL 78%, HSTCL 13% [7]. The COG-group reported an even better outcome (5-year OS 70%, EFS 60%) from the analysis of 20 pediatric patients with PTCL [9]. In contrast, the largest retrospective study of 1314 adult patients with PTCL excluding ALK + ALCL showed a 5-year OS of approximately 35% [17].

In view of the poor outcome with conventional chemotherapy, high-dose-chemotherapy and ASCT have been proposed for consolidation in adults with chemotherapy-sensitive disease following the results of many retrospective and prospective single-arm studies. Most of the studies report 5-year PFS and OS rates of 41–46%, and 50–70%, respectively for patients who received ASCT in CR1, as compared to 5-year PFS and OS rates of 20–40%, and 26–66% for those not receiving ASCT in CR1 [18–25]. The potential benefit of ASCT applies only to relatively young and fit patients with chemotherapy-sensitive PTCL. However, in many studies patients with ALCL were included, making outcome estimates for patients with non-anaplastic PTCL more difficult.

In our cohort, out of 9 patients with PTCL (8 PTCL-NOS, 1 SPTCL) receiving ASCT, 6 were in CR1 and 2 in CR2. Of those transplanted in CR, 7 were alive at last follow up. Similar results were reported by Kobayashi et al. [26] with 7 pediatric PTCL patients who underwent ASCT, 4 of them in CR1. The estimated 5-year EFS of 50% and OS of 75% was slightly better than the 5-year OS/EFS of 66.7% for patients receiving ASCT after induction failure/relapse [26]. In another series only single pediatric patients received ASCT for PTCL. Those who reached CR before ASCT remained long term disease-free [7, 8]. Notably, the number of CRs had no adverse influence on the outcome. Given the small numbers from previous studies and our current analysis it remains difficult to recommend selection criteria for consolidative ASCT in first line PTCL-therapy in childhood. It might be an option for patients with intermediate risk, whereas patients with less aggressive subtypes of PTCL and chemotherapy-sensitive disease could maintain a durable CR after conventional chemotherapy with the possibility of a rescue-ASCT in CR2. Achievement of CR prior to ASCT seems to be pivotal prognostic factor, also for children. Therefore, in addition to modern imaging techniques like PET-CT, detection of minimal residual disease should be included in assessment of the disease-status prior to ASCT.

In our analysis 16 patients were transplanted in second remission (11 in CR2, 5 in PR/VGPR2), two of them received ASCT, and 14 allo-HSCT. There were 4 deaths (2 disease-related and 2 NRM). Ten (71%) patients treated with allo-HSCT were alive and disease-free. As suggested by many investigators [3, 7, 26, 27] given the data from our study, we would agree that allo-HSCT could be offered to high-risk patients, as soon as they reach CR.

Twenty-seven patients in our study underwent allo-HSCT as consolidation after first-line therapy. Sixteen were in CR1, three in VGPR1, and eight in PR1. Of those, sixteen (59%) were alive and disease free at the time of analysis, 11 patients died (4 disease progression, 7 NRM). In the Japanese cohort 4 patients received allo-HSCT in CR1/PR1, with a reported 5-year OS of 100% [26]. In another study of 13 children with PTCL, 7 received allo-HSCT, 3 of them in CR. Of the seven patients, 3 were alive, and 4 died of NRM [8]. In another analysis allogeneic and autologous HSCT was conducted in 14 and 2 pediatric patients with PTCL-NOS, respectively, of whom 6 were in CR1, 9 in CR2, and one in PR-status. 5 and 5 of the patients transplanted in CR1 and CR2, respectively, survived, the patient transplanted in PR died [7]. The data, although limited by the small number of patients, show a good efficacy of allo-HSCT and demonstrate a graft-versus-lymphoma effect. However, the high NRM as shown also in our study hampers better outcome.

In our analysis most of the patients with allo-HSCT (87%) received MAC, with 2/3 of the conditioning being TBI-based (67,5%). Considering that veno-occlussive disease and infections were the leading causes of NRM, the high therapy burden of MAC might pose a higher risk of toxic complications. GvHD was reported to have contributed to the NRM in two cases, only.

In adults, allo-HSCT has also been used as consolidation treatment in high-risk PTCL. Compared with ASCT, allo-HSCT led to lower relapse rate, however, at cost of higher NRM [28, 29]. A randomized phase-3 study investigated autologous versus allo-HSCT as part of first-line therapy in high-risk PTCL in adults [30]. There was no significant difference in the 3-years OS between the groups. Relapses were mostly seen after ASCT, whereas NRM (31%) occurred only after allo-HSCT with MAC. This study led to the recommendation to reserve allo-HSCT for relapsed/refractory PTCL. NRM rate of 26% after allo-HSCT in CR/PR1 in our analysis was lower than described in adults, but still substantial. Allo-HSCT in CR/PR1 may thus be an option for pediatric patients with high-risk disease. Use of reduced-intensity conditioning regimen in adults showed a reduction of NRM without compromising the EFS rates [31–33]. In our study only 6 patients (13%) received non-myeloablative conditioning, all of them in CR1/PR1. 3 were alive at last follow-up, 3 died of NRM.

Reported outcomes for adults with PD or primary refractory PTCL are very poor, with a median OS of 9.1 months [34]. In our study, 6 patients were transplanted without achieving remission prior to HSCT. Five underwent allo-HSCT, four died of lymphoma after HSCT. The one patient with ASCT at PD died of NRM. In the Japanese study of 19 pediatric patients with PTCL (12 PTCL-NOS, 6 ENKTCL, 1 SPTCL) having induction failure or relapse, who underwent HSCT, the 5-year EFS and OS was 50% for PTCL-NOS patients, and EFS of 50% and OS of 66.7% for ENKTCL, respectively [26]. However, the study did not report the disease status prior to HSCT.

Our study has some limitations. Since we analyzed data from the EBMT registry, only patients receiving HSCT were included. This may have led to selection bias, since on the one hand, patients with a low-risk profile were possibly adequately treated without HSCT. On the other hand, patients with a very high-risk profile might have not reached transplantation or chosen palliative treatment. With the retrospective nature of the study there were no uniform criteria for choosing the mode of transplantation. The primary therapy varied considerably as did the conditioning regimen before HSCT, not allowing for conclusions about the optimal treatment strategies.

In conclusion, this study describes the to date largest group of pediatric patients with PTCL undergoing HSCT. It underlines the role of allo-HSCT for patients with an intermediate to high-risk profile who reach at least partial remission. Due to the rarity of this heterogenic lymphoma group in children, implementation of randomized studies is not feasible even on an international platform. Prospective register studies with defined criteria for entering autologous or allogeneic transplantation might help to find optimal treatment strategies for children with PTCL.

Our data suggest a role of allogeneic HSCT in consolidation-treatment of patients with high-risk disease, who reach at least partial remission after primary- or relapse-therapy, whereas autologous HSCT could be an option for intermediate-risk patients with complete remission. Patients with a PD status prior to transplantation did not profit from HSCT. Further biologic studies about the disease mechanisms and novel therapeutic approaches, including cellular therapies are needed for these patients.

### Supplementary information

Supplemental material

## Data Availability

The datasets generated during and/or analyzed during the current study are available from the corresponding author on reasonable request.

## References

[1] Swerdlow SH, Campo E, Harris NL, Jaffe ES, Pileri SA, Stein H, et al. WHO Classification of Tumours of Haematopoietic and Lymphoid Tissues. IARC, Lyon. (2017).

[2] Kobayashi R, Yamato K, Tanaka F, Takashima Y, Inada H, Kikuchi A (2010). Retrospective analysis of nonanaplastic peripheral T-cell lymphoma in pediatric patients in Japan. Pediatr Blood Cancer.

[3] Kontny U, Oschlies I, Woessmann W, Burkhardt B, Lisfeld J, Salzburg J (2015). Non-anaplastic peripheral T-cell lymphoma in children and adolescents—a retrospective analysis of the NHL-BFM study group. Br J Haematol.

[4] Windsor R, Stilles C, Webb D (2008). Peripheral T-cell lymphomas in childhood: population-based experience in the United Kingdom over 20 years. Pediatr Blood Cancer.

[5] Flower A, Xavier AC, Cairo MS (2019). Mature (non-anaplastic, non-cutaneous) T-/NK-cell lymphomas in children, adolescents and young adults: state of the science. Br J Haematol.

[6] Au-Yeung RK, Richter J, Abramov ID, Bacon CM, Balagué O, d’Amore ES, et al. Molecular features of non-anaplastic peripheral T-cell lymphoma in children and adolescents. Pediatr Blood Cancer. 2021. 10.1002/pbc.29285. 10.1002/pbc.29285 34390161

[7] Mellgren K, Attarbaschi A, Abla O, Alexander S, Bomken S, Bubanska E (2016). Non-anaplastic peripheral T cell lymphoma in children and adolescents-an international review of 143 cases. Ann Hematol.

[8] Al Mahmoud R, Weitzman S, Schechter T, Ngan B, Abdelhaleem M, Alexander S (2012). Peripheral T-cell lymphoma in children and adolescents: a single-institution experience. J Pediatr Hematol Oncol.

[9] Hutchison RE, Laver JH, Chang M, Muzzafar T, Desai S, Murphy S (2008). Non-anaplastic peripheral Tcell lymphoma in childhood and adolescence: a Children’s Oncology Group study. Pediatr Blood Cancer.

[10] Sandlund JT, Perkins SL (2015). Uncommon non-Hodgkin lymphomas of childhood: pathological diagnosis, clinical features and treatment approaches. Br J Haematol.

[11] Möricke A, Zimmermann M, Reiter A, Henze G, Schrauder A, Gadner H (2010). Long-term results of five consecutive trials in childhood acute lymphoblastic leukemia performed by the ALL-BFM study group from 1981 to 2000. Leukemia.

[12] Reiter A, Schrappe M, Parwaresch R, Henze G, Muller-Weihrich S, Sauter S (1995). Non-Hodgkin’s lymphomas of childhood and adolescence: results of a treatment stratified for biologic subtypes and stage-a report of the Berlin-Frankfurt-Munster Group. J Clin Oncol.

[13] Reiter A, Schrappe M, Ludwig WD, Tiemann M, Parwaresch R, Zimmermann M (2000). Intensive ALL-type therapy without local radiotherapy provides a 90% event-free survival for children with T-cell lymphoblastic lymphoma: a BFM group report. Blood.

[14] Reiter A, Schrappe M, Tiemann M, Ludwig WD, Yakisan E, Zimmermann M (1999). Improved treatment results in childhood B-cell neoplasms with tailored intensification of therapy: A report of the Berlin-Frankfurt-Münster Group Trial NHL-BFM 90. Blood.

[15] Le Deley MC, Rosolen A, Williams DM, Horibe K, Wrobel G, Attarbaschi A (2010). Vinblastine in children and adolescents with high-risk anaplastic large-cell lymphoma: results of the randomized ALCL99-vinblastine trial. J Clin Oncol.

[16] Wrobel G, Mauguen A, Rosolen A, Reiter A, Williams D, Horibe K (2011). Safety assessment of intensive induction therapy in childhood anaplastic large cell lymphoma: report of the ALCL99 randomised trial. Pediatr Blood Cancer.

[17] Vose J, Armitage J, Weisenburger D, International T-Cell Lymphoma Project. (2008). International peripheral T-cell and natural killer/T-cell lymphoma study: pathology findings and clinical outcomes. J Clin Oncol.

[18] Fossard G, Broussais F, Coelho I, Bailly S, Nicolas-Virelizier E, Toussaint E (2018). Role of up-front autologous stem-cell transplantation in peripheral T-cell lymphoma for patients in response after induction: an analysis of patients from LYSA centers. Ann Oncol.

[19] Yam C, Landsburg DJ, Nead KT, Lin X, Mato AR, Svoboda J (2016). Autologous stem cell transplantation in first complete remission may not extend progression-free survival in patients with peripheral T cell lymphomas. Am J Hematol.

[20] Ellin F, Landström J, Jerkeman M, Relander T (2014). Real-world data on prognostic factors and treatment in peripheral T-cell lymphomas: a study from the Swedish Lymphoma Registry. Blood.

[21] Abramson JS, Feldman T, Kroll-Desrosiers AR, Muffly LS, Winer E, Flowers CR (2014). Peripheral T-cell lymphomas in a large US multicenter cohort: prognostication in the modern era including impact of frontline therapy. Ann Oncol.

[22] Mehta N, Maragulia JC, Moskowitz A, Hamlin PA, Lunning MA, Moskowitz CH (2013). A retrospective analysis of peripheral T-cell lymphoma treated with the intention to transplant in the first remission. Clin Lymphoma Myeloma Leuk.

[23] Wilhelm M, Smetak M, Reimer P, Geissinger E, Ruediger T, Metzner B (2016). First-line therapy of peripheral T-cell lymphoma: extension and long-term follow-up of a study investigating the role of autologous stem cell transplantation. Blood Cancer J.

[24] d’Amore F, Relander T, Lauritzsen GF, Jantunen E, Hagberg H, Anderson H (2012). Up-front autologous stem-cell transplantation in peripheral T-cell lymphoma: NLG-T-01. J Clin Oncol.

[25] Corradini P, Tarella C, Zallio F, Dodero A, Zanni M, Valagussa P (2006). Long-term follow-up of patients with peripheral T-cell lymphomas treated upfront with high-dose chemotherapy followed by autologous stem cell transplantation. Leukemia.

[26] Kobayashi R, Fujita N, Mitsui T, Iwasaki F, Suzumiya J, Kuroda H (2012). Stem cell transplantation for paediatric patients with non-anaplastic peripheral T-cell lymphoma in Japan. Br J Haematol.

[27] Attarbaschi A, Abla O, Padilla LA, Beishuizen A, Burke GA, Brugières L (2020). Rare non-Hodgkin lymphoma of childhood and adolescence: A consensus diagnostic and therapeutic approach to pediatric-type follicular lymphoma, marginal zone lymphoma, and nonanaplastic peripheral T-cell lymphoma. Pediatr Blood Cancer.

[28] Mollee P, Lazarus HM, Lipton J (2003). Why aren’t we performing more allografts for aggressive non-Hodgkin’s lymphoma?. Bone Marrow Transpl.

[29] Le Gouill S, Milpied N, Buzyn A, De Latour RP, Vernant JP, Mohty M (2008). Graft-versus lymphoma effect for aggressive T-cell lymphomas in adults: a study by the Societe Francaise de Greffe de Moelle et de Therapie Cellulaire. J Clin Oncol.

[30] Schmitz N, Truemper L, Bouabdallah K, Ziepert M, Leclerc M, Cartron G (2021). A randomized phase 3 trial of autologous vs allogeneic transplantation as part of first-line therapy in poor-risk peripheral T-NHL. Blood.

[31] Corradini P, Dodero A, Zallio F, Caracciolo D, Casini M, Bregni M (2004). Graft versus-lymphoma effect in relapsed peripheral T-cell non-Hodgkin’s lymphomas after reduced-intensity conditioning followed by allogeneic transplantation of hematopoietic cells. J Clin Oncol.

[32] Castagna L, Pagliardini T, Bramanti S, Schiano de Colella JM, Montes de Oca C, Bouabdallah R (2021). Allogeneic stem cell transplantation in poor prognosis peripheral T-cell lymphoma: the impact of different donor type on outcome. Bone Marrow Transpl.

[33] Zhang JY, Briski R, Devata S, Kaminski MS, Phillips TJ, Mayer TL (2018). Survival following salvage therapy for primary refractory peripheral T-cell lymphomas (PTCL). Am J Hematol.

[34] Huang H, Jiang Y, Wang Q, Guo L, Jin Z, Fu Z (2017). Outcome of Allogeneic and Autologous Hematopoietic Cell Transplantation for High-Risk Peripheral T Cell Lymphomas: A Retrospective Analysis From a Chinese Center. Biol Blood Marrow Transpl.

