# Supplementary material for: Hematopoietic stem cell transplantation for pediatric patients with non-anaplastic peripheral T-cell lymphoma. An EBMT pediatric diseases working party study
Source: Bone Marrow Transplant. 2024 Feb 8;59(5):604–14. doi: 10.1038/s41409-024-02226-1 (PMC11073963; doi:10.1038/s41409-024-02226-1)
Supplement: Supplementary file 1 — Supplemental material [file 41409_2024_2226_MOESM1_ESM.docx]

Supplementary material:

Items included in the questionaire sent to the participating centres for additional information not available from the EBMT database

| Patient identification | UPN  Treating centre  Country of the centre  Patient following national /international study (yes, no, unknown)?  If yes, name of the study |
| --- | --- |
| Patient characteristics | Date of birth  Gender of the patient  Date of diagnosis  Diagnosis NHL/PTCL  Subtype of PTCL  Size of the largest mass (<5cm, 5-10cm, >10cm, not measurable, no mass, unknown)  LDH level (normal, elevated, unknown)  Known cancer predisposition syndrome  Known immune deficiency  If yes, please specify  Stage of disease at diagnosis (I, II, III, IV, unknown)  Systemic symptoms (A, B, unknown)  Specific sites of involvement (CNS, bone marrow, nodes above diaphragma, nodes below diaphragma, liver, intestinal tract, lung, skin, other) |
| Treatment before HSCT | Treatment pre-HSCT (yes, no, unknown)  Primary therapy (T-NHL-type, B-NHL-type, other)  Date of first-line therapy  Drugs used for first-line therapy  Radiotherapy (yes, no, unknown)  Response to primary treatment (CR, PR>50%, SD, relapse/progression, never in CR, NA, unknown)  Relapse after first-line treatment (yes, no, unknown)  Date of relapse  Secondary therapy (no therapy, T-NHL-type, B-NHL-type, other)  Drugs used for second-line therapy  Radiotherapy (yes, no, unknown)  Response to secondary treatment (CR, PR>50%, SD, relapse/progression, never in CR, NA, unknown)  Relapse after second-line treatment (yes, no, unknown)  Number of relapses prior to HSCT  CR achieved prior tot he first HSCT? |
| Status at first HSCT | Disease status at first HSCT (CR, PR>50%, SD, relapse/progression, never in CR, NA, unknown)  Number of this status (1st, 2nd, 3rd or higher, unknown)  Date of first transplantation  Type of first HSCT (allogeneic, autologous)  Source of stem cells (bone marrow, peripheral blood, cord blood)  Donor type, allogeneic HSCT only (syngeneic, identical sibling, other matched relative, mismatched relative, metched unrelated, mismatched unrelated, NA)  Graft manipulation ex vivo (yes, no, unknown) |
| Conditioning | Regimen intended to be myeloablative (yes, no,unknown)  TBI (yes, no, unknown)  TBI total dose (Gy)  Radiotherapy other than TBI (yes, no, unknown)  Cyclophosphamide (yes, no, unknown)  Busulfan (yes, no, unknown)  Treosulfan (yes, no, unknown)  Etoposide (yes, no, unknown)  Fludarabine (yes, no, unknown)  Thiotepa (yes, no, unknown)  Melphalan (yes, no, unknown)  Cytarabine (yes, no, unknown)  BCNU (yes, no, unknown)  Dexamethasone (yes, no, unknown)  Other (please specify) |
| GvHD-Prophylaxis | ATG (yes, no, unknown)  ALG (yes, no, unknown)  Campath (yes, no, unknown)  Corticosteroids (yes, no, unknown)  Cyclophosphamide (yes, no, unknown)  Ciclosporine (yes, no, unknown)  Methotrexate (yes, no, unknown)  Mycophenolate mofetil (yes, no, unknown)  Other (please specify) |
| After HSCT | Hematologic recovery/engraftment (engrafted, no engraftment, graft loss, NA, unknown)  Acute GvHD maximum grade (no aGVHD, grade I, grade II, grade III, grade IV, present-grade not known, not known)  Chronic GvHD (yes, no, unknown)  Extent of cGvHD (limited, extended, not known)  Status of cGvHD at last follow-up (no, resolved, continuous since last reported episode, recurrence)  Best response within 100 days following the first HSCT (CR, PR>50%, SD, relapse/progression, never in CR, NA, unknown)  Relapse after first HSCT (yes, no, unknown)  Date of relapse  Status at last follow-up (alive, dead, lost to follow-up)  Date of last follow-up  Main cause of death (HSCT-related, relapse or progression of original disease, secondary malignancy, other, uknown, NA) |

Abbreviations: aGvHD: acute graft-versus-host disease, cGvHD: chronic graft-versus-host disease, CR: complete remission, NA: not applicable, PR: partial remission, SD: stable disease, TBI: total body irradiation, UPN: unique patient number
